# Force, impulse and energy during falling with and without knee protection: an in-vitro study

**DOI:** 10.1038/s41598-019-46880-8

**Published:** 2019-07-17

**Authors:** Michael Schwarze, Christof Hurschler, Bastian Welke

**Affiliations:** Laboratory for Biomechanics and Biomaterials, Department of Orthopaedics, Hannover Medical School, 30625 Hannover, Germany

**Keywords:** Biomedical engineering, Orthopaedics

## Abstract

The mechanics of protective knee padding mitigating injury from a high-force fall have not been investigated in real-life scenarios to date. This study compares the effect of wearing knee pads to unprotected impact on a hard surface. We hypothesized that knee pads reduce the force and energy transmitted to the bony structures of the knee cap compared with unprotected conditions. Eight human knee cadaver specimens were embedded and fixed with a flexion angle of 100 degrees in a custom-made drop testing device (75 kg including the knee). The usage of a knee pad led to an average peak force attenuation on impact of 15% (no pad: 5932 N SD: 2472 N; pad: 4210 N SD: 2199 N; p < 0.001). Contact time on the plate was higher with a knee pad (no pad: 0.015 s SD: 0.009 s; pad: 0.028 s SD: 0.014 s; p < 0.001). Therefore, the observed impulse was also increased (no pad: 62.2 Ns SD: 17.8 Ns; pad: 74.6 Ns SD: 18.6 Ns; p < 0.001). This effect diminished as drop height was increased. Energy dissipation, defined as the difference between kinetic energy pre-impact and peak potential energy post-impact, was higher without a knee pad (no pad: 10.5 J SD: 6.2 J; pad: 4.2 J SD: 5.0 J; p < 0.001). The results from this study illustrate the magnitude of influence that knee pads have on peak forces, transmitted impulse, and energy transfer from a high-force impact in real-life scenarios. Contrary to expectations, the knee pad did not act as a mechanical damper. The mechanical behavior more closely resembled a spring that temporarily stores energy and consequentially reduces peak forces upon impact. Based on this study, future developments in padding might benefit from focusing on the aspect of energy storage and temporarily delayed energy dissipation.

## Introduction

Impact on the knees during a fall is a hazard prevalently occurring during various sporting activities, or in the elderly community^1,2^. In some sports, the usage of protective equipment, such as pads, is mandatory. To reduce the risk of injury, padding must reduce the impact forces acting on the musculoskeletal system. This can be achieved by either damping (i.e. dissipation of energy), or temporarily storing energy. For athletes, the use of kneepads results in a 56% reduction in the rate of knee injuries^3^. In the elderly, special padding on the hips can reduce the effect of an unexpected fall^4^. In addition to preventing major fractures, a reduction in local pressure on the impact zone is also desirable. For simple pads, a proportional relationship was found between maximum acceleration, which is directly proportional to force, and peak pressure^5^.

In the real world, a hazardous load is induced via a short impulse after conversion of potential energy while standing to kinetic energy during a fall^6^. Previous literature, concerning the fracture of human femurs as a result of falling, conducted biomechanical tests in a quasi-static state and fracture was induced by force^7^. A few studies that used high-velocity impacts had a very high incidence of bone fracture, from 27% with a crushing interface to 100% with rigid contact^8,9^. The aim of our study was to find a dynamic load scenario between these extremes which mimic loads during falling, and does not necessarily lead to a fracture.

A standard for protective padding was established several years ago^10^. Although this standard allows for comparison among different pads, it does not provide a benchmark reference that includes data from human tissue. The design of our study attempts to reflect loading conditions on the knee that were previously observed in gait analysis and numerical simulation studies^11,12^.

Currently, only very limited data exist on the mechanics of protecting pads in high-force scenarios. In this study, the effect of wearing knee pads is compared to an unprotected impact on a hard surface. The results of the presented study can serve as a reference when evaluating protective padding materials. We hypothesized that knee pads reduce the force and energy transmitted to the bony structures of the knee cap compared with unprotected conditions.

## Methods

For ethical reasons, tests evaluating the effectiveness of knee padding should not be performed with live participants, or trials need to be limited in force and energy to minimize pain and fracture risk to the participants^4,5^. A part from numerical simulation, *in-vitro* testing with cadaver specimens also allows the carrying out of high-force impact studies in a controlled environment. Previous studies focused more on subchondral damage after impact loading, by utilizing porcine, canine, or leporine models^13–16^, which cannot be applied directly to conditions in human subjects and differ in focus.

Eight cadaveric human knee specimens were obtained from six donors (Science Care, Phoenix, Arizona, USA). The donors, or authorized representatives, gave informed consent for the use of these specimens in scientific research. Mean age of the donors was 68.3 years and their mean weight was 77.3 kg. Available medical records on the donors did not report any pathology in the lower extremities. Ethical approval was given by the local Ethics Committee of Hanover Medical School, under the lead of Professor Tröger (approval No. 1320-2012), and experiments were performed in accordance with relevant guidelines and regulations. Before preparation, the knee specimens were thawed at room temperature overnight. After the specimens thawed, the skin and subcutaneous soft tissue were removed at the proximal and distal end, leaving the muscles, articular capsule, tendons, and ligaments intact. The tibia and femur were embedded centrally into a metal shell using cold-curing, three-component resin (Rencast FC52/53 Isocyanate, FC53 Polyol, DT82 Filler, Huntsman Corporation, The Woodlands, USA). The femur and tibia had a minimum residual length of 170 mm outside of the embedded section. The patella was held in place by applying tension to the quadriceps tendon via an embedded wire. The amount of force was adjusted manually to ensure firm positioning of the patella.

**Figure 1:**
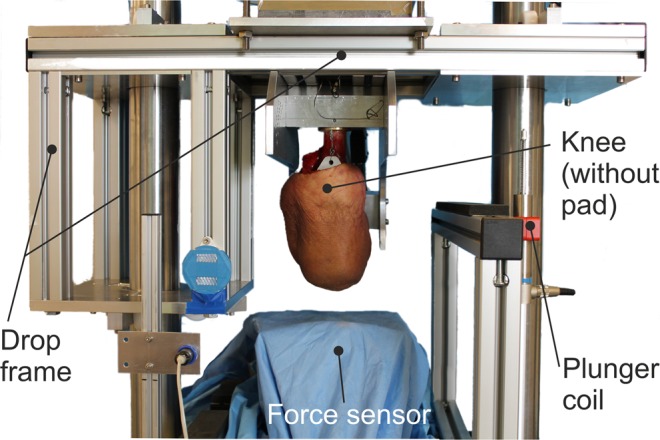
Custom made drop testing device for human knee specimen (mounted to MTS MiniBionix II, Model 858, Eden Prairie. Minneapolis).

The knee specimens were fixed with the femur perpendicular to the impact surface at a flexion angle of 100 degrees in a custom-made drop testing device. The drop testing device was mounted onto a servohydraulic material testing machine (MTS MiniBionix II, Model 858, Eden Prairie, Minneapolis), as shown in Fig. 1. The flexion angle was chosen to replicate the physiologic impact angle for a knee during a fall^12^. The testing device had a total mass of 75 kg including the knee specimen, resembling the body mass of an average adult.

A commercially available knee pad (Oxygen 2, Thailand) with a hard polyethylene (PE) shell and a soft foam insert was attached to the knee for the protective padding portion of the testing. This knee pad was chosen to replicate the protective conditions in a previously conducted study which evaluated a healthy participant subjected to various falling scenarios^12^. The exact mechanism of protection was not known and will be one of the results from this study. In this study, specimens were lifted 10, 20, 30 and 40 mm from a fully resting position on a force sensor and subsequently released to fall along guiding rods onto a rigid and level aluminum plate. Each height was tested once per specimen and condition, with the padded condition first. Falling height was determined a-priori during pre-tests with one additional knee specimen, in order to have measured forces correspond to realized forces of a real-life fall^11,12^. Force and falling speed were continuously monitored at 1,000 Hz. The falling speed was obtained by differentiating position data from a plunger coil. Rebound height of the knee was calculated from the lowest position at impact to the maximum bounce height.

Peak force was recorded in each trial and shows the maximum force in the trial from the initial impact and not the subsequent force after bouncing. The impulse is the integral of force and time from the beginning of a trial until the force has dropped below 50% of its maximum value. This percentage of maximum value was chosen because it is applicable to all trials unedited. Duration of force was defined according to a referenced study^12^, as the time during which measured force was above 50% of the peak force. Kinetic energy was defined as E_pre_ = 0.5 mv^2^ (m: mass and v- velocity of the drop frame) and potential energy as E_post_ = mgh (g: gravitational constant and h: rebound height). Energy dissipation was defined as the difference between maximum kinetic energy pre-impact and maximum potential energy post-impact.

## Statistics

Determination of sample size (n = 8) was done before beginning the testing and was based on established protocols for *in-vitro* tests, as well as budget limitations. Statistical comparison was performed by paired Wilcoxon tests, with a significance level of alpha = 0.05, where applicable. Regression analysis was conducted by fitting a linear model for the response (i.e. peak force, impulse, potential energy after impact, and energy dissipation) over a predictor variable (i.e. speed and kinetic energy before impact). For comparison to real-life scenarios, the authors had access to the source data of Welke *et al*.^12^.

## Results

A total of 64 tests were conducted. For various reasons, a few trials (n = 4) could not be completely processed: specimen number 13 was not tested in the 10 mm falling height (n = 2); and data from specimens number 3 and number 10 could not be processed due to low impact force and speed in the padded 10 mm drop height condition (n = 2).

**Figure 2:**
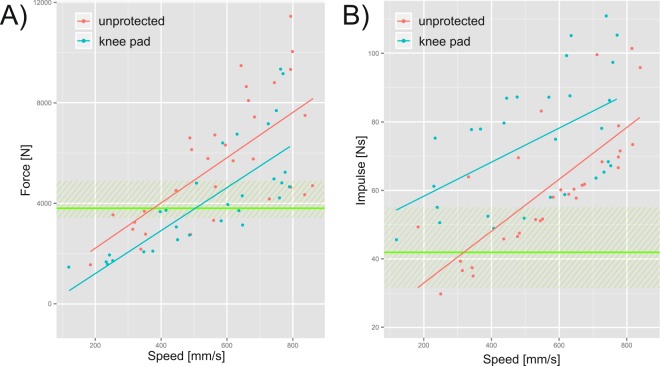
(**A**) Peak force plotted over peak falling speed. (**B**) Impulse plotted over peak falling speed. Trials without a knee pad are colored in red, trials including a knee pad are colored in blue. The range of values observed during forward falling and landing on one knee of human subjects are shaded in green, and the average is indicated as a green line^12^.

Abiding by the laws of physics, higher falling heights were associated with higher falling speeds. Peak impact forces ranged from 1,800 N to 11,600 N and there was a linear dependence to falling speed (regression’s R²_pad_ = 0.60 and R²_unprotected_ = 0.45). The usage of a knee pad lead to a significant attenuation of peak forces on impact by an average of 15% (Fig. 2a) compared to the unprotected condition (p < 0.001). The measured forces were generally in a range similar to the forces observed during real-life falling scenarios, with the more extreme conditions exceeding them by a factor of four^12^.

**Figure 3:**
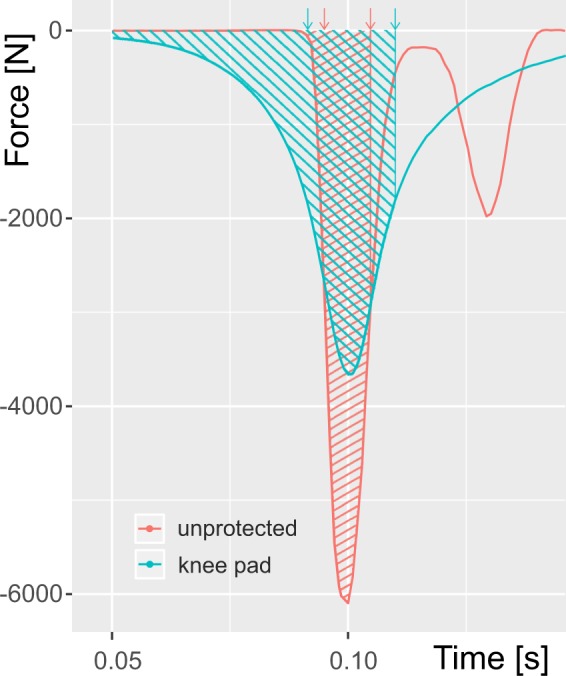
Force plotted over time in a typical trial. The falling height was 30 mm and the unprotected condition is shown in red and the pad condition in blue. The impulse as calculated from the force –time integral used for analysis is highlighted as a shaded area for both conditions. Duration of force as calculated according to^12^ and is indicated by arrows in corresponding colors. Both plots are aligned to have their force peak at t = 0.1 s for comparison.

The duration of contact in trials with a knee pad was significantly longer than in unprotected knee trials (pad: 26.9 ms, SD: 14.4 ms; unprotected: 15.0 ms, SD: 8.9 ms; p < 0.001). Therefore, the observed impulse was significantly increased (p < 0.001, Fig. 2b, example: Fig. 3). The difference in impulse, between the padded and unprotected conditions, diminished with increasing falling speed.

**Figure 4:**
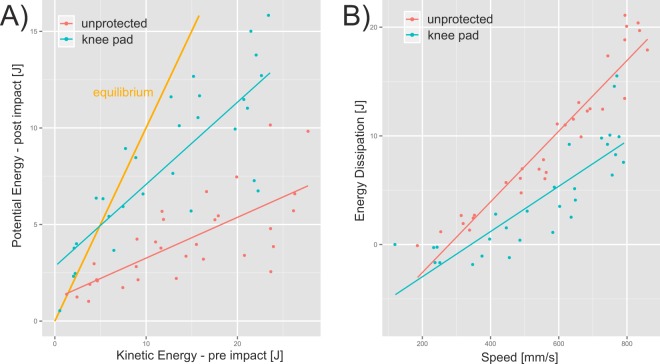
(**A**) Peak potential energy of the drop testing device after impact plotted over peak kinetic energy before the impact. Equilibrium is highlighted in yellow. (**B**) Differences between both energies (i.e. dissipation) plotted over peak falling speed. Trials without a knee pad are colored in red; trials including a knee pad are colored in blue.

Energy dissipation showed a strong linear dependence on falling speed (regression’s R²_pad_ = 0.71 and R²_unprotected_ = 0.91) and was significantly higher without a knee pad (p < 0.001, Fig. 4a,b). The difference in energy dissipation between the padded and unprotected condition was negligible at low falling speeds, up to 400 mm/s with energy below 2 J, while increasing to over 7 J with falling speeds over 600 mm/s.

A fracture of the bone was observed in 2 out of 8 specimens, via x-ray examination carried out after testing. Both were distal femoral fractures originating between the condyles. In one case, the fracture continued spirally through the diaphysis and in the other case it ended after 6 cm. No microcracks were visible in any of the conducted x-ray examinations.

## Discussion

The results from this study illustrate the magnitude of influence that knee pads have on peak forces, transmitted impulse, and energy transfer from a high-force impact in real-life scenarios. In the design of our study, we tried to achieve highly dynamic loads on the knee, as previously observed in a gait analysis and numerical study, and further exceed those loads^11,12^. It was important that duration of the loads was in the range of 10 ms. Therefore an actuator driven test, such as with a servohydraulic test machine, was ruled out. The load on the femur is applied by first converting potential energy to kinetic energy, which leads to a force acting over a specific amount of time, known as an impulse. This impulse loads the specimen, the resulting rebound possessing kinetic energy, and finally potential energy at the apex of the rebound. During pre-tests, neither using the same falling height as observed in reality (roughly 450 mm), nor using the same falling speed (up to 800 mm/s) were considered to be appropriate matching parameters because our rigid set-up did not flex in the hip, as a human would when falling on the ground^12^. Hence, we decided to match the *in-vitro* setup to the previous study on acting force and impulse which resulted in falling heights between 10 mm to 40 mm.

As desired, values for forces were comparable to a previous inverse dynamics study by Welke *et al*., where forward falling of a healthy subject was investigated^12^. Reviewing the source data of Welke *et al*., we found 61% of the measured impulses were higher than the impulses observed during real-life scenarios. The range of the real-life impact impulse was 32 Ns to 55 Ns, while observations in the preset study ranged between 25 Ns and 106 Ns.

Contrary to expectations, the knee pad did not act as a mechanical damper. The mechanical behavior more closely resembled a spring that temporarily stored energy, reduced peak forces, and prolonged the duration of impact.

Our tests can be classified as ranging from elastic to fracture (at least in two cases) according to Gupta *et al*.^17^. As the specimens cover this whole range, we could expect to have some specimens in the continuum damage range. However, we could not observe any corresponding microcracks in the standard x-rays because of limited resolution. Both fractures are likely attributed to the shear forces occurring when one condyle begins transmitting load to the diaphysis, while the other is not in contact with the surface yet.

A study conducted by Hoshino and Wallace tested the impact absorbing properties of the knee in a fully extended position, which was closer to a landing model and slightly different from the current falling model^18^. Their focus was to investigate the effect of a total knee replacement on peak impact forces compared to an intact knee. While their reported forces were much lower than those in the presented study (intact knee: average of 1598 N), their reported durations of contact were also lower (intact knee: ~2 ms). These two effects combined and led to a reduced impulse.

A study performed by Atkinson and Haut investigated the effect of different knee flexion angles on forces and fractures after impact by one rigid and one deformable body^8^. They found lower peak forces (mean peak force for deformable and rigid impact: Atkinson: 5.3 kN; our study: 7.3 kN) and higher impact energies compared to the highest falling height in our study (mean energy for deformable and rigid impact: Atkinson: 31.1 J; our study: 23.1 J) for a 90 degree flexion angle. According to Atkinson, the flexion angle has a significant effect on impact energy, with peak forces and temporal impact parameters with a 120-degree flexion angle having the highest loads. It should be noted that their deformable surface is not comparable to the knee pad in the presented study, since it is made of a crushable aluminum honeycomb structure. They found a high number of gross macrofractures with a rigid impact (n = 18/18) and concluded that an energy of 28 J was needed to produce a fracture. With a crushing force impact, a lower number of fractures (n = 5/18) was observed and the energy required was found to be 56 J. However, they did not calculate the energy dissipated by the bone.

The major limitation of this drop setup is the high-rigidity of the drop frame and the mounting method of the knee. In natural falling where the individual is unable to stop the fall with their hands, the hip would flex and consequentially reduce forces during impact. An additional limitation related to the experimental setup is the fact that the specimens do not reach their theoretical falling speeds and speeds between specimens varies. This is explained by friction in the setup and a non-ideal release of the drop test frame. The effect is more pronounced at lower falling heights. Another noted limitation is only the first peak in measured forces was evaluated. Subsequent forces as a result from oscillation of the knee on the plate were neglected. The authors regard this procedure as valid, because subsequent forces were comparatively lower than the first peak and therefore unlikely to be responsible for damaging the bone. As another limitation, the forces generated in the drop impact can be considered as very high compared to forces observed in protective equipment testing. In standardized testing of protective equipment, mean forces shall not exceed 6 kN^10^. This however does not prevent such high forces from occurring in reality.

In the measurements, some outliers belonging to the protected knees are present that appear to violate the principle of energy conservation, since post-impact potential energy was higher than the pre-impact kinetic energy at lower speeds. This is due to calculation of the pre-impact energy being based on maximum falling speed. In the case of protected knees, at the time of maximum speed, potential energy is not completely converted to kinetic energy because the knee pad slows down the falling and energy is stored. Therefore, the pre-impact energy is underestimated in the protected knees. The last noted limitation is only one type of knee pad was used in the trials. There are certainly other protective devices available on the market, but the majority consist of similar technology, using foam as a shock absorber. Comparing different protective pads was beyond the scope of this study.

## Conclusion

This study revealed the observed reduction in peak force, transmitted impulse, and energy absorption from falling onto a knee that is padded compared to an unprotected knee. The pad behaves like a mechanical spring, which temporarily stores energy. Further research should include the influence of the pad material and shape. Additionally, the ideal protection mechanism (e.g. reduced peak force, reduced impulse, increased energy dissipation) for the knee should be investigated. The application of high-frequency pressure sensors would explain the extent to which the contact area of the impact increases with protection. The ideas presented in this study might aid in developing advanced concepts for hazard-reducing padding utilized in an automotive interior, or crash test dummies^19^.

## Data Availability

The datasets generated during and/or analysed during the current study are available from the corresponding author on reasonable request.

## References

[1] Nugent R (1974). Protective equipment in amateur sport. Can. Fam. physician Médecin Fam. Can..

[2] Kotschwar, A. & Peham, C. Comparison of Single- and Multilayer Material used as Dampening Elements in Knee-Protectors. In *ISBS-Conference Proceedings Archive* (2009).

[3] Yang J (2005). Use of discretionary protective equipment and rate of lower extremity injury in high school athletes. Am. J. Epidemiol..

[4] Laing AC, Tootoonchi I, Hulme PA, Robinovitch SN (2006). Effect of compliant flooring on impact force during falls on the hip. J. Orthop. Res..

[5] Laing AC, Robinovitch SN (2008). Effect of soft shell hip protectors on pressure distribution to the hip during sideways falls. Osteoporos. Int..

[6] Cordey J, Schneider M, Bühler M (2000). The epidemiology of fractures of the proximal femur. Injury.

[7] Cheng XG (1997). Assessment of the strength of proximal femur *in vitro*: relationship to femoral bone mineral density and femoral geometry. Bone.

[8] Atkinson PJ, Haut RC (2001). Impact Responses of the Flexed Human Knee Using a Deformable Impact Interface. J. Biomech. Eng..

[9] Hayashi, S., Choi, H.-Y., Levine, R. S., Yang, K. H. & King, A. I. Experimental and Analytical Study of Knee Fracture Mechanisms in a Frontal Knee Impact. In, 10.4271/962423 (1996).

[10] Protective clothing - Wrist, palm, knee and elbow protectors for users of roller sports equipment - Requirements and test methods; German version EN 14120:2003 + A1:2007 (2007).

[11] Schwarze M, Hurschler C, Seehaus F, Correa T, Welke B (2014). Influence of transfemoral amputation length on resulting loads at the osseointegrated prosthesis fixation during walking and falling. Clin. Biomech..

[12] Welke B, Schwarze M, Hurschler C, Calliess T, Seehaus F (2013). Multi-body simulation of various falling scenarios for determining resulting loads at the prosthesis interface of transfemoral amputees with osseointegrated fixation. J. Orthop. Res..

[13] Lahm A (2005). An experimental canine model for subchondral lesions of the knee joint. Knee.

[14] Newberry WN, Mackenzie CD, Haut RC (1998). Blunt impact causes changes in bone and cartilage in a regularly exercised animal model. J. Orthop. Res..

[15] Radin EL, Paul IL, Lowy M (1970). A comparison of the dynamic force transmitting properties of subchondral bone and articular cartilage. J. Bone Joint Surg. Am..

[16] Ewers BJ, Weaver BT, Sevensma ET, Haut RC (2002). Chronic changes in rabbit retro-patellar cartilage and subchondral bone after blunt impact loading of the patellofemoral joint. J. Orthop. Res..

[17] Gupta HS, Zioupos P (2008). Fracture of bone tissue: The ‘hows’ and the ‘whys’. Med. Eng. Phys..

[18] Hoshino A, Wallace WA (1987). Impact-absorbing properties of the human knee. J. Bone Jt. Surgery, Br. Vol..

[19] King AI (2001). Fundamentals of impact biomechanics: Part 2–Biomechanics of the abdomen, pelvis, and lower extremities. Annu. Rev. Biomed. Eng..

